# Anti-depressant medication use is a risk factor for incident knee surgery and opioid use in patients with knee osteoarthritis

**DOI:** 10.3389/fpsyt.2023.1243124

**Published:** 2023-11-27

**Authors:** Yongqiang Zheng, Jinshan Zhang, Zefeng Wang, Xiaofeng Liu, Yongquan Xu, Yangzhen Fang, Zhenyu Lin, Liang Lin

**Affiliations:** Department of Orthopedics, Jinjiang Municipal Hospital, Fujian, China

**Keywords:** KOA, anti-depressant, risk factor, knee surgery, opioid, depression

## Abstract

**Objective:**

To investigate whether depression and exposure to anti-depressant medication are independent risk factors for incident knee surgery and opioid use in knee osteoarthritis (KOA) patients.

**Methods:**

We identified all patients who visited our outpatient department and were clinically diagnosed with KOA between January 2010 and January 2018. We retrieved their demographic, clinical, and radiographic data from the database of our hospital. Next, we analyzed the effect of depression and anti-depressant medication on the incident knee surgery and opioid use in KOA patients.

**Results:**

A total of 4,341 KOA patients were found eligible to form the study population. Incident knee surgery and opioid use for the purpose of treating osteoarthritis were observed in 242 and 568 patients, respectively. Incident knee surgery was significantly associated with age (OR [95%CI], 1.024 [1.009–1.039], *P* = 0.002), BMI (OR [95%CI], 1.090 [1.054–1.128], *P* < 0.001), baseline K-L grade 3 (OR [95%CI], 1.977 [1.343–2.909], *P* = 0.001), baseline K-L grade 4 (OR [95%CI], 1.979 [1.241–3.157], *P* = 0.004), depression (OR [95%CI], 1.670 [1.088–2.563], *P* = 0.019), and exposure to anti-depressant medication (OR [95%CI], 2.004 [1.140–3.521], *P* = 0.016). Incident opioid use was significantly associated with depression (OR [95%CI], 1.554 [1.089–2.215], *P* = 0.015) and exposure to anti-depressant medication (OR [95%CI], 1.813 [1.110–2.960], *P* = 0.017).

**Conclusion:**

Depression and anti-depressant drug exposure were independently associated with incident knee surgery, highlighting the need for more attention on comorbid depression in KOA management.

## Introduction

Knee osteoarthritis (KOA) is a prevalent and disabling disease characterized by the structural degradation of cartilage, persistent pain, joint stiffness, and functional limitations, affecting more than 10% of the overall population as estimated (1–3). According to the Global Burden of Disease Study 2019 (4), because of the increase in the aging population, the disease burden of KOA has been rapidly growing worldwide over the past decades (5).

Depression is also a major challenge for medical society with rapidly increasing disease burden in recent decades (6). Studies conducted in different countries consistently showed that depression is greatly more prevalent among patients with osteoarthritis (7–9). Thus, there is a huge population affected by depression and osteoarthritis simultaneously. A previous study showed that more than 20% of patients with osteoarthritis had depressive symptoms (10). Notably, a recent study revealed a huge care gap in this large population that only half of KOA patients with comorbid depression received mental health (11). Conversely, depression largely contributed to rapid progression (8, 12). Therefore, the interplay between KOA and depression and depression-related medications should be investigated with urgency (13).

Opioids are recommended by multiple guidelines for the management of KOA pain after failure of other pain-relieving medications (14, 15). However, the misuse and abuse of opioid medications is an emerging challenge over the past two decades (16). Many factors are associated with this issue, including aggressive pain control and inappropriate prescription (17). Both depression and osteoarthritis were deeply involved in this opioid abuse/misuse epidemic (18–20). Opioid exposure in KOA patients contributed to increased medical cost, impaired productivity, potential for abuse, and increased mortality (19, 21, 22). Therefore, reducing prolonged opioid use may lead to a meaningful reduction in social and economic costs for KOA patients (19). However, depressed patients are at substantial risk of prolonged opioid use (18, 23). In addition, the severity of depression is closely associated with opioid-related problems (24). Opioid use itself also contributed to new onset and progression of depression (25, 26).

The aims of this study were, therefore, to determine whether depression and exposure to anti-depressant medications were associated with the incidence of knee surgery and opioid use.

## Patients and methods

### Study population

We identified all patients who visited our outpatient department and were clinically diagnosed as KOA between January 2010 and January 2018 using the hospital information system (HIS). The clinical diagnosis of KOA was made by clinical specialists in orthopedic and/or sports medicine. It was generally determined based on patient history, physical examination, and laboratory and radiographic findings (27). Patients were excluded from this study if they had structural knee injuries (fractures, ligament ruptures, meniscal tears, and dislocations) (*n* = 345), any knee surgery histories (*n* = 125), missing values for baseline variables (*n* = 567), and declined to participate (*n* = 1,152). Our study adhered to the Declaration of Helsinki and all local laws and regulations, and we obtained ethics approval for collecting all related data.

### Baseline patient data

To better assess the radiographic severity of KOA, we defined the baseline as the time of performing the first knee plain radiograph during the study period. All baseline demographic and clinical data were retrieved from HIS, and the information was further confirmed by contacting patients by all possible means. The baseline demographic data consisted of demographic information [age, sex, body mass index (BMI)] and education. The Kellgren–Lawrence (K-L) grades (28) were rated by a radiographic evaluation committee consisting of three radiologists specialized in musculoskeletal radiology. The rating process was conducted without grouping information. The consensus on grading was achieved by the majority. When the two knees had different K-L grades, the final K-L grade was recorded according to the more severe side. Generally, in our institution, we treated KOA patients initially with topical or oral NSAIDs. When the initial treatment fails, we would recommend intra-articular therapies and opioid medication based on the preferences of patients.

### Depression and anti-depressant drugs

The diagnosis of depression and exposure to anti-depressant drugs, including selective serotonin reuptake inhibitors (SSRIs), tricyclic anti-depressants (TCAs), and serotonin-norepinephrine reuptake inhibitors (SNRI), were first reported by patients and further confirmed by reviewing their medical records.

### Definition of incident knee surgery and opioid exposure

The incident knee surgery and types of surgery were reported by patients. The incident knee surgery was defined as any surgical procedure performed to treat KOA, no matter whether this type of surgical procedure was recommended or not. Incident knee surgery in the current study included knee arthroplasty, arthroscopic procedures, and high tibial osteotomy (HTO). The efficacy and safety of arthroplasty and HTO have been well-established and generally recommended for KOA management (29, 30). However, although many high-quality randomized trials have consistently demonstrated that arthroscopic procedures, including lavage, debridement, and arthroscopic partial meniscectomy, are ineffective and even harmful for KOA patients by accelerating joint destruction and increasing future arthroplasty risks, these high-quality evidence most published in leading medical journals have failed to prevent surgeons from performing arthroscopic procedures for managing KOA (31–34). We, therefore, also considered incident arthroscopic procedures as clinically important events of poor symptom control and disease progression. Incident opioid exposure was defined as the initiation of opioid use during the study period for opioid-naïve patients at baseline. The opioid exposure history was reported by patients and confirmed by reviewing their medical records by researchers.

### Statistical analysis

All statistical analyses were performed using SPSS software (26.0, IBM, Chicago, USA). The statistical significance was set at a two-sided 0.05. We first tabulated descriptive statistics to summarize the characteristics of the subjects. Continuous and categorical variables were, respectively, presented as means ± standard deviations and counts (percentage), unless otherwise indicated. When the yielded *P*-value was less than 0.2 in univariable analysis, the variables along with demographic variables (age, sex, and BMI) were further included in logistic regression for multivariable analysis.

## Results

### The association between depression/anti-depressant medication and incident knee surgery

A total of 4,341 KOA patients were found eligible for the final analysis. Incident knee surgery to treat osteoarthritis was observed in 242 patients. Specifically, 70 patients had knee arthroplasty, 162 had arthroscopic procedure, and 10 had high tibial osteotomy. Of the 179 patients with exposure to anti-depressant medication, 146 patients used these drugs for their depressive disorders, 20 for Alzheimer's disease, 6 for obsessive-compulsive disorder, 4 for phobia, and 3 for other mental disorders. For univariable analyses, incident knee surgery was significantly associated with age, BMI, baseline K-L grades, depression, and exposure to anti-depressant medication (Table 1). For multivariable analysis, incident knee surgery was significantly associated with age (OR [95%CI], 1.024 [1.009–1.039], *P* = 0.002), BMI (OR [95%CI], 1.090 [1.054–1.128], *P* < 0.001), baseline K-L grade 3 (OR [95%CI], 1.977 [1.343–2.909], *P* = 0.001), baseline K-L grade 4 (OR [95%CI], 1.979 [1.241–3.157], *P* = 0.004), depression (OR [95%CI], 1.670 [1.088–2.563], *P* = 0.019), and exposure to anti-depressant medication (OR [95%CI], 2.004 [1.140–3.521], *P* = 0.016; Table 2).

**Table 1:** Univariable analysis on characteristics grouped by incident knee surgery.

	**No incident knee surgery (*n* = 4,099)**	**Incident knee surgery (*n* = 242)**	***P-*value**
Age, years	61.02 ± 8.83	62.93 ± 8.46	< 0.001
**Sex, No. (%)**			
Male	1,068 (26.1%)	70 (28.9%)	0.324
Female	3,031 (73.9%)	170 (71.1%)	
Baseline BMI, kg/m^2^	25.07 ± 3.59	26.26 ± 4.39	< 0.001
**Depression, No. (%)**			
Yes	381 (9.3%)	44 (18.2%)	< 0.001
No	3,718 (90.7%)	198 (81.8%)	
**Anti-depressant medication exposure, No. (%)**			
Yes	155 (3.8%)	24 (9.9%)	< 0.001
No	3,944 (96.2%)	218 (90.1%)	
**Hypertension, No. (%)**			
Yes	1,335 (32.6%)	86 (35.5%)	0.339
No	2,764 (67.4%)	156 (64.5%)	
**K-L grades, No. (%)**			
0 or 1	1,134 (27.7%)	48 (19.8%)	0.001
2	1,766 (43.1%)	96 (39.7%)	
3	833 (20.3%)	66 (27.3%)	
4	366 (8.9%)	32 (13.2%)	
**Diabetes, No. (%)**			
Yes	505 (12.3%)	32 (13.2%)	0.678
No	3,594 (87.7%)	210 (86.8%)	
**Smoking, No. (%)**			
Yes	357 (8.7%)	24 (9.9%)	0.519
No	3,742 (91.3%)	218 (90.1%)	
**Education, No. (%)**			
More than 9 years	1,036 (25.3%)	66 (27.3%)	0.488
No more than 9 years	3,063 (74.7%)	176 (72.7%)	

Data are shown as means (±SD) unless otherwise indicated. BMI, body mass index; K-L, Kellgren-Lawrence.

**Table 2:** Multivariable analysis on characteristics grouped by incident knee surgery.

	**Odds ratio (95% Confidence interval)**	***P*-value**
Baseline BMI	1.090 (1.054–1.128)	< 0.001
Age	1.024 (1.009–1.039)	0.002
Depression (Reference: No)	1.670 (1.088–2.563)	0.019
Anti-Depressant medication (Reference: No)	2.004 (1.140–3.521)	0.016
K-L grade 2 (Reference: K-L grade 0 or 1)	1.797 (0.924–1.892)	0.127
K-L grade 3 (Reference: K-L grade 0 or 1)	1.977 (1.343–2.909)	0.001
K-L grade 4 (Reference: K-L grade 0 or 1)	1.979 (1.241–3.157)	0.004

The logistic regression model included baseline sex, BMI, age, depression, anti-depressant medication, and K-L grades.

### The association between depression/anti-depressant medication and incident opioid use

A total of 2,227 KOA patients were opioid-naïve and thus included in the subsequent analysis. During the study period, 568 patients initiated opioid therapies. For univariable analyses, incident opioid use was also significantly associated with depression and exposure to anti-depressant medication (Table 3). For multivariable analysis, incident opioid use was significantly associated with depression (OR [95%CI], 1.554 [1.089–2.215], *P* = 0.015) and exposure to anti-depressant medication (OR [95%CI], 1.813 [1.110–2.960], *P* = 0.017; Table 4).

**Table 3:** Univariable analysis on characteristics grouped by incident opioid use in opioid-naïve KOA patients.

	**No incident opioid use (*n* = 1,659)**	**Incident opioid use (*n* = 568)**	***P-*value**
Age, years	60.91 ± 8.80	61.05 ± 8.25	0.744
**Sex, No. (%)**			
Male	442 (26.6%)	150 (26.4%)	0.913
Female	1,217 (73.4%)	418 (73.6%)	
Baseline BMI, kg/m^2^	25.21 ± 3.73	25.10 ± 3.51	0.525
**Depression, No. (%)**			
Yes	128 (7.7%)	79 (13.9%)	< 0.001
No	1,531 (92.3%)	489 (86.1%)	
**Anti-depressant medication exposure, No. (%)**			
Yes	53 (3.2%)	43 (7.6%)	< 0.001
No	1,606 (96.8%)	525 (92.4%)	
**Hypertension, No. (%)**			
Yes	572 (34.5%)	180 (31.7%)	0.225
No	1,087 (65.5%)	388 (68.3%)	
**K-L grades, No. (%)**			
0 or 1	457 (27.5%)	152 (26.8%)	0.968
2	711 (42.9%)	242 (42.6%)	
3	348 (21.0%)	123 (21.7%)	
4	143 (8.6%)	51 (8.9%)	
**Diabetes, No. (%)**			
Yes	206 (12.4%)	69 (12.1%)	0.866
No	1,453 (87.6%)	499 (87.9%)	
**Smoking, No. (%)**			
Yes	140 (8.4%)	50 (8.8%)	0.789
No	1,519 (91.6%)	518 (91.2%)	
**Education, No. (%)**			
More than 9 years	431 (26.0%)	138 (24.3%)	0.427
No more than 9 years	1,228 (74.0%)	430 (75.7%)	

Data are shown as means (± SD) unless otherwise indicated. BMI, body mass index; K-L, Kellgren-Lawrence.

**Table 4:** Multivariable analysis on characteristics grouped by incident opioid use in opioid-naïve KOA patients.

	**Odds ratio (95% Confidence interval)**	***P*-value**
Depression (Reference: No)	1.554 (1.089–2.215)	0.015
Anti-depressant medication (Reference: No)	1.813 (1.110–2.960)	0.017

The logistic regression model included baseline sex, BMI, age, depression, anti-depressant medication, and K-L grades.

## Discussion

Although more than 20% of patients with osteoarthritis had depressive symptoms (10), depression is frequently underdiagnosed and undertreated (35). Thus, in this study, the prevalence of clinically diagnosed depression in this study was lower than 10%. This fact also once again argued that comorbid depression in KOA patients needed more attention from physicians and patients. A previous study revealed that pain symptoms caused by KOA would largely contribute to the progression of depression (36). Conversely, patients with depressive disorder have substantial pain over-sensitization in the central nervous system, which leads to more severe symptoms in KOA patients (37). A pilot study using a combined approach to address both depression and KOA simultaneously yielded more benefits for improving depression, pain, and functional outcomes (13). In addition, we also demonstrated that depression increased the chance of opioid use in KOA patients. We all know inappropriate prescription of opioids played an important role in the opioid epidemic. The misuse rate of overprescribed opioids was strikingly high, highlighting the urgency of restraining opioid use in KOA patients. Careful management of depression could help to curb the opioid epidemic. Notably, depressed patients initiate opioid therapy slightly more often than non-depressed patients and are twice as likely to transition to long-term use (18). In studies that carefully control for confounding by indication, it has been shown that long-term opioid therapy increases the risk of incident, recurrent, and treatment-resistant depression (18). Thus, it is also important to understand the interplay between opioid use and depression in KOA patients.

Overweight/obesity is a well-established risk factor for both KOA and depression (36). For KOA management, weight management has previously been recommended as a core approach for achieving long-term benefits, especially for those with overweight/obesity (38–40). For depression, high BMI is a risk factor for the development, progression, and recurrence of depression (41, 42). Thus, weight loss might be beneficial for patients with both diseases. However, weight loss greater than 5% was considered clinically relevant, but this goal is generally difficult to reach even after exercise and dieting (43). Glucagon-like peptide-1 receptor agonists (GLP-1RAs), including semaglutide, liraglutide, and dulaglutide, are a newly emergent class of weight-reducing drugs (44). A multicenter prospective study revealed that GLP-1RAs acted as disease-modifying drugs for KOA patients mediated by weight loss (2). Future research may need to pay more attention to this class of game-changing drugs.

The current study had several limitations. First, future prospective studies are still needed to validate our findings. Notably, because of the retrospective nature, the patient-reported outcomes were unknown in this study. Second, because of the observational nature of this study, the decision process on surgical treatment and opioid use is a black box for us. Similarly, the current study could not evaluate the disease status of depression. Third, the arthroscopic procedures were ineffective and even harmful to KOA patients. The rationale behind this practice (performing arthroscopic procedures in KOA patients) remained unclear for the current study. We assumed that these receiving arthroscopic procedures reflect the poor control of symptoms caused by KOA. Finally, there is no consensus regarding the diagnosis of depression. We used clinically diagnosed depression, and extrapolation of our conclusion to a different setting should be cautious.

## Data availability statement

The raw data supporting the conclusions of this article will be made available by the authors, without undue reservation.

## Ethics statement

The studies involving humans were approved by the Ethics Committee at Jinjiang Municipal Hospital (jjsyyyxll-2012001). The studies were conducted in accordance with the local legislation and institutional requirements. Written informed consent for participation in this study was provided by the participants' legal guardians/next of kin.

## Author contributions

JZ developed the idea of the study, participated in its design and coordination, and drafted the manuscript. YZ participated in the design of the study and revised the manuscript. YX and YF assessed the radiographs and collected the data. ZL, ZW, and YZ followed up the patients, collected the data, and analyzed the data. LL and XL contributed to the acquisition and interpretation of data. All authors read and approved the final draft.
